# Global knowledge mapping and emerging trends in research between spasmolytic polypeptide-expressing metaplasia and gastric carcinogenesis: A bibliometric analysis from 2002 to 2022

**DOI:** 10.3389/fcimb.2022.1108378

**Published:** 2023-01-27

**Authors:** Lin Liu, Yang Wang, Yukun Zhao, Wei Zhang, Jiong Liu, Fengyun Wang, Ping Wang, Xudong Tang

**Affiliations:** ^1^ Institute of Digestive Diseases, Xiyuan Hospital of China Academy of Chinese Medical Sciences, Beijing, China; ^2^ Institute of Basic Theory for Chinese Medicine, China Academy of Chinese Medical Sciences, Beijing, China; ^3^ Department of Pathology, Xiyuan Hospital of China Academy of Chinese Medical Sciences, Beijing, China

**Keywords:** spasmolytic polypeptide-expressing metaplasia (SPEM), gastric carcinogenesis (SGC-7901), helicobacter pylori infection (H. pylori infection), gastric precancerous lesions, bibliomeric analysis, visualization

## Abstract

**Background:**

Spasmolytic polypeptide expression metaplasia (SPEM) occurs in the corpus of the stomach and is closely related to inflammations caused by *H. pylori* infection. Recently, SPEM was suggested as one of the dubious precancerous lesions of gastric cancer (GC). Thus, further research on SPEM cell transdifferentiation and its underlying mechanisms could facilitate the development of new molecular targets improving the therapeutics of GC. Using bibliometrics, we analyzed publications, summarized the research hotspots and provided references for scientific researchers engaged in related research fields.

**Methods:**

We searched the Web of Science Core Collection (WoSCC) for publications related to SPEM-GC from 2002 to 2022. The VOSviewer, SCImago, CiteSpace and R software were used to visualize and analyze the data. Gene targets identified in the keyword list were analyzed for functional enrichment using the KEGG and GO databases.

**Results:**

Of the 292 articles identified in the initial search, we observed a stable trend in SPEM-GC research but rapid growth in the number of citations. The United States was the leader in terms of quality publications and international cooperation among them. The total number of articles published by Chinese scholars was second to the United States. Additionally, despite its low centrality and average citation frequency, China has become one of the world’s most dynamic countries in academics. In terms of productivity, Vanderbilt University was identified as the most productive institution. Further, we also observed that Gastroenterology was the highest co-cited journal, and Goldenring Jr. was the most prolific author with the largest centrality.

**Conclusion:**

SPEM could serve as an initial step in diagnosing gastric precancerous lesions. Current hotspots and frontiers of research include SPEM cell lineage differentiation, interaction with *H. pylori*, disturbances of the mucosal microenvironment, biomarkers, clinical diagnosis and outcomes of SPEM, as well as the development of proliferative SPEM animal models. However, further research and collaboration are still required. The findings presented in this study can be used as reference for the research status of SPEM-GC and determine new directions for future studies.

## 1 Introduction

According to the World Health Organization, gastric cancer (GC) was the third leading cause of cancer-related deaths worldwide in 2020 (Smyth et al., 2020). Before developing into GC, the gastric mucosa undergoes pathological changes such as gastritis, atrophy, intestinal metaplasia (IM) and atypical hyperplasia (Correa et al., 1975). However, there is a limited understanding on the etiology and pathogenesis of GC, which contributes to its poor prognosis (Ajani et al., 2017). Previous studies showed that several factors, particularly Helicobacter pylori (*H. pylori*) infection, can contribute to abnormal gastric mucosal cell differentiation (Mills and Shivdasani, 2011). It is often characterized by chronic inflammation followed by parietal cell defects, which can accelerate the occurrence and development of GC (Wroblewski et al., 2010). Two types of metaplastic GC are related to parietal cell defects in the gastric corpus: IM, characterized by intestinal-type cells, and antral spasmolytic polypeptide metaplasia (SPEM), characterized by trefoil factor 2 (TFF2) in deep antral glands (Saenz and Mills, 2018). Previous studies using animal models confirmed that SPEM induced by *H. pylori* infection in mice progressed only to dysplasia, not IM, suggesting that SPEM could be the beginning of a precancerous process (Weis and Goldenring, 2009; Hibdon and Samuelson, 2018; Goldenring and Mills, 2022). In contrast to IM, the origin, regulatory mechanism and link to the pathogenesis of GC for SPEM are still unknown (Nomura et al., 2004; Yoshizawa et al., 2007; Nam et al., 2009; Nam et al., 2010). In addition, endoscopic and pathological findings on SPEM are relatively insidious, restricting related clinical observation and treatment because it is hard to diagnose. As a result, knowing the origin and progression mechanisms of SPEM could help prevent it from progressing into dysplasia or GC. Thus, comprehensive research on SPEM-GC is necessary to assist scientists in obtaining deeper insights into the trends in SPEM-GC related research.

Bibliometrics is a mathematical and statistical approach to analyze research literature (Kokol et al., 2021) and was defined by Prof Pritchard (1969) as aiming to discover the patterns of scientific literature in a specific field. Although various methods can be used to perform a quantitative overview, from traditional and systematic reviews to main path analyses and evidence maps, only bibliometrics contribution and cooperation of authors, institutions, countries, journals and keywords can provide qualitative and quantitative forecasts of hotspots and trends in certain research topics (van Eck et al., 2010; Chen, 2017; Liu et al., 2022). On the other hand, bibliometric mapping can be used to visualize the structure and patterns of research literature production in the form of science landscapes (Kokol et al., 2018). In this regard, although SPEM has recently gained the spotlight of the research community, a bibliometric analysis of SPEM-GC has not yet been reported. Thus, this study aimed to quantify the entire picture of SPEM research in the last 20 years using bibliometric software and R packages, which might contribute to generating suggestions for future research about SPEM and GC.

## 2 Methods

### 2.1 Database and study collection

**Figure 1:**
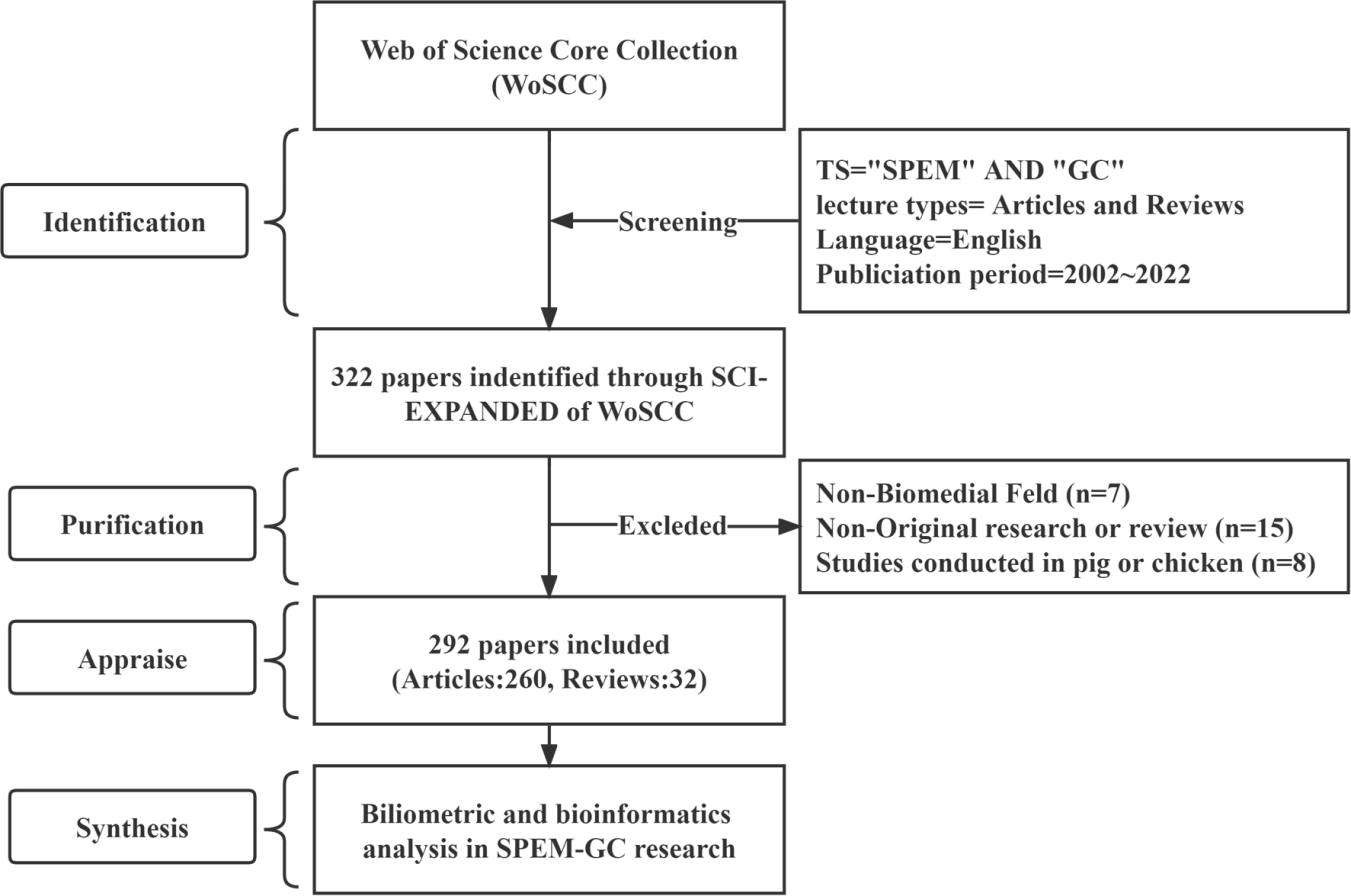
Literature search and screening flowchart.

According to previous bibliometric studies, the Web of Science Core Collection (WoSCC) is the most widely used database, with more than ten thousand high-quality journals (Liu et al., 2022; Zhang et al., 2022a; Zhang et al., 2022b). A database from the WoSCC, the Science Citation Index-Expanded, was selected to conduct this study. The literature search for original articles and reviews was conducted independently by two researchers on October 1, 2022, using the search terms: #1: Topic (TS)= (spasmolytic polypeptide-expressing metaplasia OR trefoil factor 2 OR TFF2); #2: TS= (gastric cancer OR gastric carcinoma OR stomach cancer OR stomach neoplasm*); Source of final search term: #1 AND #2. The study period was from January 1, 2002, to October 1, 2022, with English used as the only language. In total, 260 articles and 32 reviews were obtained (**Figure 1**). **Supplementary File S1** displays the included studies.

### 2.2 Data compilation and purification

A range of key information was extracted from the WoSCC database for further analysis in this study. This series contained information such as the year of publication, the number of citations, countries or regions, research organizations, authors, sources, references and keywords. Then, several repetitive keywords, such as nations, organizations, authors and keywords, were combined into one word, spelling errors were corrected, and irrelevant words were removed. Lastly, the bibliometric analysis was completed by importing the cleaned data into Microsoft Excel 365 and bibliometric visualization platforms.

### 2.3 Analyses and visualizations

A nation’s productivity is generally measured by the number of publications, and its impact is measured by the number of average citations. In addition, screening procedure based on Price’s Law ( $\sum_{m+1}^{I}n\left(x\right)=\sqrt{N}$ ), was employed in this study to identify representative scholars and key research forces. In this equation, x denoted the number of publications from each author and n (x) denoted the number of authors who have written x number of publications. Additionally, I equal to nmax represented the number of documents produced by the highest-producing author, N represented the total number of authors, and m represented the minimum number of documents produced by the core author. As a result, $m=\mathrm{0.749}\times \sqrt{N_{max}}\approx \mathrm{5.3}$ was used to define authors with ≥6 documents issued as the core author (Knowlson et al., 2022).

Diagrams of visualizing network and knowledge structure were conducted using VOSviewer, a widely used bibliometric visualization software (van Eck and Waltman, 2010), which provides three main visual maps: the network visualization map, time-overlay visualization map and density visualization map. This study used VOSviewer (Version 1.6.18) to analyze co-authorship between countries, organizations, core authors, influential journals and co-occurrence keywords (Kokol et al., 2018). Furthermore, SCImago Graphica (Version 1.0.26) was primarily used to assess the international geographic collaborations and distribution among the top ten productive countries (Hassan-Montero et al., 2022). Additionally, CiteSpace (Version 6.1.R3), another visualization tool invented by Prof. Chaomei Chen (Synnestvedt et al., 2005), was also used in this study to visualize the co-citation analysis of references and authors and identify the keywords and references with the strongest citation bursts. CiteSpace was also used to create a dual map overlay of journals using parameters: duration (2002–2022), years sliced (3 years), the type of node (reference, cited author, and cited journal), selection criteria (g-index = 25), and pruning methods (Pathfinder and pruning sliced networks). An additional Cytoscape plugin, CytoNCA, was used to conduct centrality analyses among the productive countries, organizations and authors of SPEM-GC.

### 2.4 GO and KEGG annotations

To further review the key pathways and targets related to the regulation of SPEM transformation, GO classification and KEGG pathway (http://www.omicsbean.cn/) methods were employed to identify genes’ functional categories and predict their biological functions. Figures were generated with R using KEGG pathway enrichment analysis and GO enrichment analysis with a corrected p-value < 0.05.

## 3 Results

### 3.1 Trend analysis of annual publications and citations

**Figure 2:**
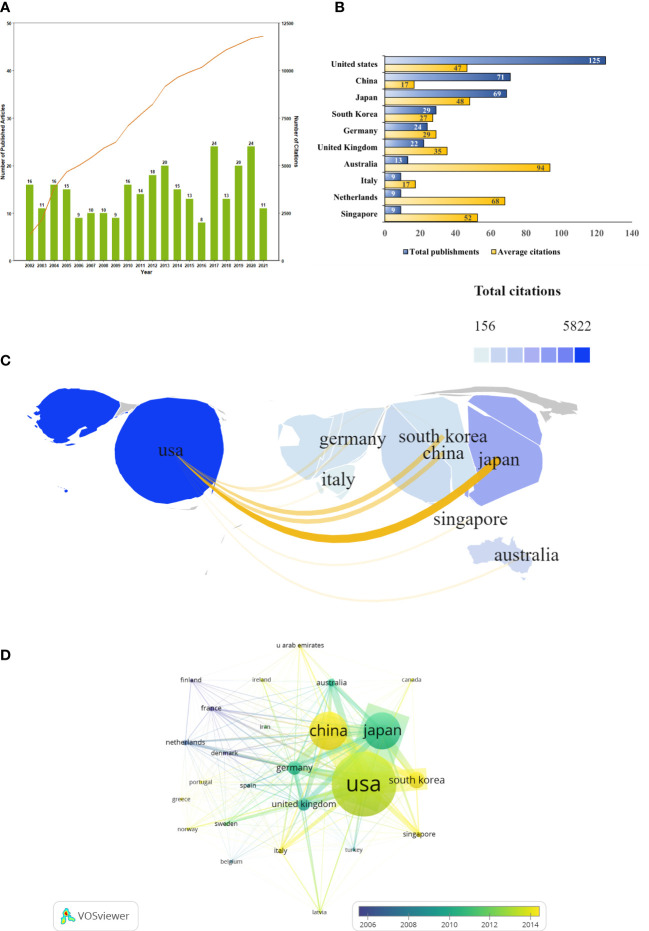
SPEM-GC publications trends and co-occurrence map over the last two decades. **(A)** Global trend in publication outputs and total citations per year on SPEM-GC from 2002 to 2022. **(B)** Core countries/regions of publications and average citations on SPEM-GC from 2002 to 2022. **(C)** The global geographic collaborations and distribution visualization map ranked the top ten countries/regions in terms of the total number of documents issued. The size of the map area represents the weight of the number of documents issued by the state accounting for the total number of documents issued, with a larger area suggesting more documents issued. The color of the map represents the total citation of articles published in each country, with a darker color representing a greater number of total citations. Yellow lines represent the intensity of joint documents issued between different regions, with wider lines representing a greater number of cooperative documents issued by the two. **(D)** Visualization of the citation overlay map for core countries/regions. In the node group, nodes represent countries and regions, and their size shows the number of publications. The connections between nodes represent the interrelation between citations. The width of the links reveals the citation strength.

A total of 292 SPEM-GC papers written by 1757 authors at 412 organizations from 41 countries were collected. They were published in 131 journals, cited by 1395 journals and referenced in 8431 articles. The total number of citations was 9345, and the average number of citations per publication was 35.76. The H-index of all the documents was 53, indicating a high academic value and social impact for papers in this field. Based on **Figure 2A**, we observed that the number of documents issued each year from 2002 to 2012 was relatively stable, except for two small peaks in 2017 and 2020. In contrast, the annual citation curve increased steadily since 2002 and peaked in 2021 when 11,816 citations were recorded.

### 3.2 Contribution of core-productive countries and organizations

To determine which countries had the most prominent contribution and cooperation in the field of SPEM-GC, we made a visual analysis of the number of documents issued, citations, and co-occurrence frequencies for 41 countries and regions. The results showed that most SPEM-GC studies were from North America, Europe and East Asia. The number of publications of the top 10 productive countries/regions continued to rise rapidly (**Figure 2B**). As shown in **Table 1**, the top ten productive countries/regions had the highest number of publications. The United States published 125 papers in this field, accounting for 40.7% of all papers. Although China ranked second in terms of publications, it had the lowest average citation among the top 10 productive countries.

**Table 1:** The top 10 productive countries involved in SPEM-GC research.

Rank	Country	Publications	Citations	Average Citation	Centrality
1	United States	125	5822	46.6	0.47
2	China	71	1174	16.5	0.29
3	Japan	69	3313	48.0	0.06
4	South Korea	29	789	27.2	0
5	Germany	24	696	29.0	0.35
6	United Kingdom	22	777	35.3	0.32
7	Australia	13	1217	93.6	0.03
8	Italy	9	156	17.3	0.19
9	Netherlands	9	612	68.0	0.06
10	Singapore	9	472	52.4	0.16

Co-authorship network maps of the top 10 productive countries were generated using VOSviewer and SCImago (**Figure 2C**). The results showed that the United States was a leading cooperation center in this field, with close ties to Japan, South Korea and China. According to **Table 2**, the United States was the most central country (0.47), with Germany and the United Kingdom ranking second (0.35) and third (0.32), respectively. However, a visualization of the number and the year of issuance using VOSviewer indicated that despite European countries such as Finland and France being the first countries to publish the first research in the field, since 2014, Asian countries such as China, South Korea and Singapore have gradually become the main research centers in this field (**Figure 2D**).

**Table 2:** The top 10 productive organizations involved in SPEM-GC research.

Rank	Organization	Publications	Citations	Average Citation	Centrality
1	Vanderbilt Univ	52	3168	60.9	0.39
2	Tokyo Univ	25	1983	79.3	0.14
3	Washington Univ	21	1059	50.4	0.16
4	Michigan Univ	13	385	29.6	0.01
5	MIT	11	1449	131.7	0.05
9	Massachusetts Univ	10	1422	142.2	0.02
7	Natl Canc Ctr	10	613	61.3	0.08
8	Seoul Natl Univ	10	514	51.4	0.04
6	Columbia Univ	10	373	37.3	0.04
10	Harvard Univ	9	452	50.2	0.05

### 3.3 Contribution of active authors

**Figure 3:**
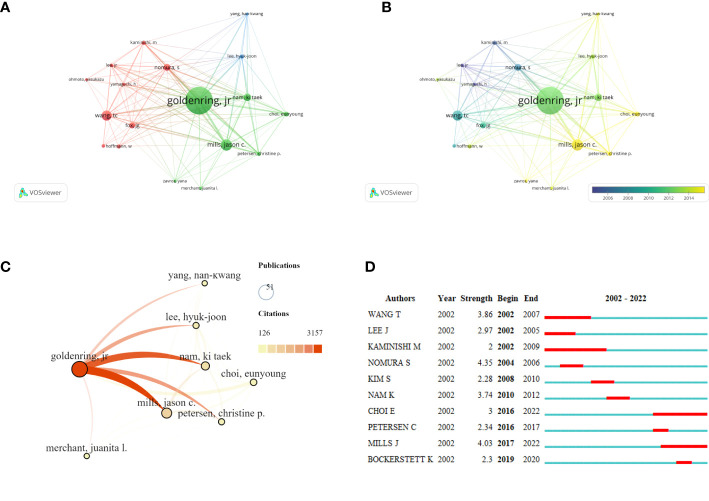
Co-occurrence analysis of active authors and their contributions. The network visualization map **(A)** and overlay visualization map **(B)** of the core co-authorship analysis generated by VOSviewer. The minimum number of documents of an author is ≥ 6. **(C)** The visualization map of the core author co-cited network carried on SCImago. **(D)** A list of the top 10 authors with the strongest citation bursts using SPEM-GC cells. Blue bars indicate the authors’ first article published, and red bars indicate the strength of the citation burst.

Based on Price’s Law, 69.7% of the total articles in this field were published by 18 core authors. More than 50% of the documents issued by core authors defined by Price’s law were evaluated, and the results suggested that the SPEM-GC research field was relatively stable in terms of authorship cooperatives. **Table 3** shows the top ten authors, organizations and countries with the most recently published papers in this field. Among them, seven of the top ten authors were from the United States, two were from South Korea, and one was from Japan. In terms of high productivity, the top three authors with the most papers were Americans. They are Goldenring, James R. Vanderbilt University (N = 51, APC = 61.9), Mills, Jason C. Washington University (N = 21, APC = 49.7), and Wang, Tc, Columbia University (N = 19, APC = 90.5). With a centrality of 0.18, Goldenring Jr. was the most cited co-author. **Figure 3A** shows the authors with a minimum of 6 publications. Based on overlay visualization of co-authorships (**Figure 3B**), the blue cluster is considered a pioneering group for SPEM-GC research. Comparatively, the yellow and Laurel-green cluster authors have published papers in recent years. Upon further visual analysis of the co-cited network, Goldenring James R. authored most of the cited articles along with Mills Jason C., Nam Ki Taek and Petersen Cheritine P. (**Figure 3C**). In terms of burst monitoring of authors (**Figure 3D**), Wang Tc, Lee Hyuk-Joon and Kaminishi M were the top three ranked institutions between 2002 and 2009, followed by Nam and Ki Taek bursting between 2010 and 2012. Presently, the bursting of Choi Eunyoung from 2016 to 2022 and Mills Jason C. indicates their dominance in this field.

**Table 3:** The top 10 productive authors involved in SPEM-GC research.

Rank	Author	Publications	Average Citation	H-Index	Centrality	Affiliation	Country
1	Goldenring, Jr	51	61.9	76	0.18	Vanderbilt Univ	United States
2	Mills, Jason C.	21	49.7	48	0.03	Baylor College of Medicine	United States
3	Wang, Tc	19	90.5	79	0.02	Columbia Univ	United States
4	Nam, Ki Taek	14	51.2	36	0	Yonsei Univ	South Korea
5	Nomura, S	13	123.8	57	0	Tokyo Univ	Japan
6	Fox, Jg	11	131.7	109	0.08	Massachusetts Univ	United States
7	Choi, Eunyoung	9	23.6	13	0.04	Vanderbilt Univ	United States
8	Petersen, Christine P.	8	34.0	17	0	Vanderbilt Univ	United States
9	Kaminishi, M	8	39.9	51	0	Vanderbilt Univ	United States
10	Lee, Hyuk-joon	8	55.1	54	0	Seoul Natl Univ	South Korea

### 3.4 Analysis of influential journals

**Figure 4:**
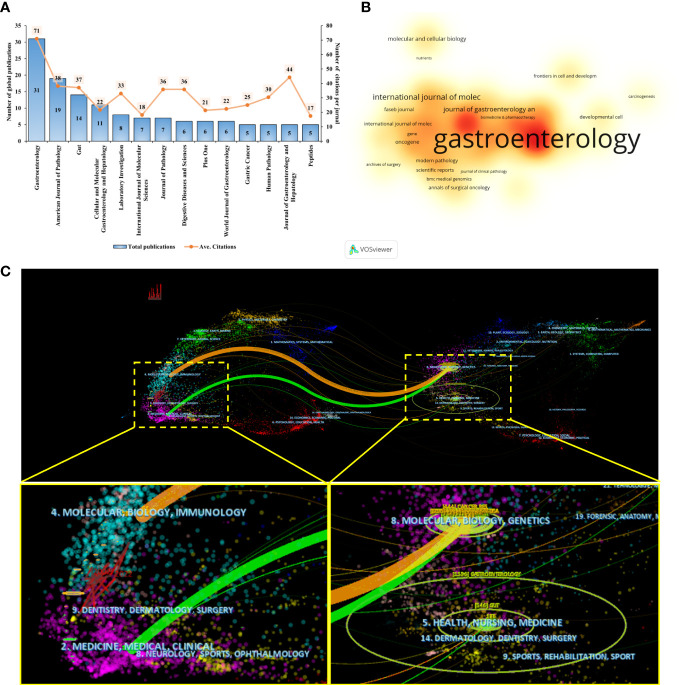
Visualization analysis of influential journals. **(A)** The publications output and average citation of the top 10 journals in SPEM-GC. **(B)** VOSviewer was used to visualize the spectral density map of journals. A deeper color indicates a greater number of citations. **(C)** CiteSpace’s dual-map overlap of SPEM-GC journals. The citing journals are on the left, and the cited journals are on the right. The colored paths indicate their citation relations.

We found that articles on SPEM-GC were published in 131 journals. As shown in **Figure 4A**, 135 documents were published in the top 14 journals (>5 documents in counts), accounting for 44% of the articles included. A total of 31 articles were published in Gastroenterology (IF 2021 = 33.9), followed by the American Journal of Pathology (IF 2021 = 5.8) and Gut (IF 2021 = 31.8). **Figure 4B** illustrates the number of citations, in which a deeper orange color indicates more co-citations. As shown in **Table 4**, most of the top ten JCI district journals with the highest average citation rate in this field were renowned journals over the past 20 years, among which 9 had an H-index over 50. The top journals in gastroenterology were Gastroenterology and Gut, Cancer Research in oncology, and Journal of Pathology in pathology. Among them, the United States and the United Kingdom had four journals each, while Japan and Switzerland had one each.

**Table 4:** The top 10 JCR Q1 Journals involved in SPEM-GC research.

Rank	Journals	Country	Average Citation	2021 IF	H-Index	Subdiscipline	Publisher	OA
1	Gastroenterology	United States	70.9	33.9	423	Gastroenterology & Hepatology	W.B. Saunders Ltd	No
2	Cancer Research	United States	46.3	13.3	466	Oncology	AACR	No
3	American Journal of Pathology	United States	38.2	5.8	289	Pathology	Elsevier	No
4	Gut	United Kingdom	37.1	31.8	311	Gastroenterology & Hepatology	BMJ	No
5	Journal of Pathology	United Kingdom	35.9	9.9	193	Oncology/Pathology	Wiley	No
6	Laboratory Investigation	United Kingdom	33.0	5.5	155	Biochemistry, Genetics and Molecular Biology/Medicine	Nature	No
7	Gastric Cancer	Japan	25.0	7.7	87	Gastroenterology & Hepatology/Oncology	Springer	No
8	Cellular and Molecular Gastroenterology and Hepatology	United States	21.6	8.8	48	Gastroenterology & Hepatology	Elsevier	No
9	International Journal of Molecular Sciences	Switzerland	18.1	6.2	195	Biochemistry, Genetics and Molecular Biology	MDPI	Yes
10	Alimentary Pharmacology and Therapeutics	United Kingdom	18.0	9.5	186	Gastroenterology & Hepatology/Pharmacology & Pharmacy	Wiley	No

Using a dual map overlay of relevant journals, we visualized the journal’s citation relationships within related fields (**Figure 4C**). In the map, the labels indicate the journal’s field. Its left side represents cited literature, and its right side represents cited literature. We determined the causal relationship between citations by determining the citation path. Citations made up the applied research and the research basis in this field. The colors signify different citation paths, with orange representing one citation path and green representing another. The orange line indicates that the included articles were mostly associated with Molecular, Biology, and Immunology disciplines. Based on the green path, the articles included in the analysis were found to be mostly distributed in Medicine, Medical and Clinical fields, while their cited papers were mostly distributed in Molecular, Biology, and Genetics.

### 3.5 Analysis of highly citing and co-cited references

**Figure 5:**
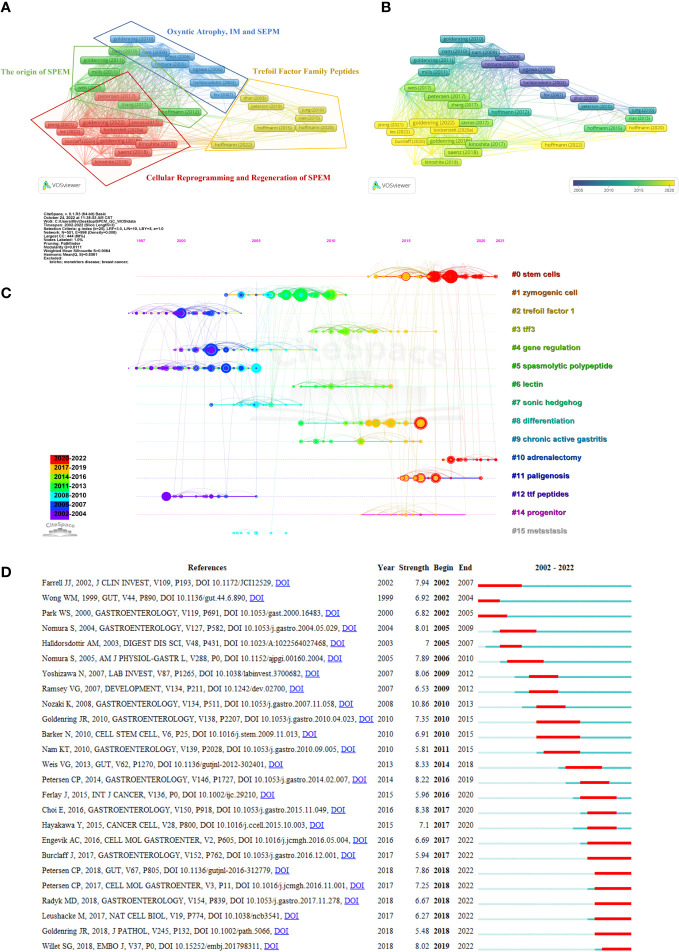
Coupling and co-citation analysis of references. **(A)** Top 50 highly citing references cluster map. The node’s size represents the strength of the references, and the link indicates the correlation between them. Each node is colored according to the cluster in which the two papers cited the same reference more frequently. **(B)** Time-overlay visualization map of the top 50 highly cited references. **(C)** The timeline shows all the co-cited references related to SPEM-GC. Each horizontal line represents a cluster, and #0 represents the largest cluster. Co-cited frequencies are reflected in node size, and links indicate co-citation relationships. The color of the node and line represent different years. The nodes represent their first co-citation. **(D)** Graph showing the top 25 references with the greatest citation bursts involved in SPEM-GC (sorted by beginning year). The blue bars indicate that the reference has been published, and the red bars represent how citations have burst during the studies’ publication.

Reference analysis was conducted, and VOSviewer and CiteSpace were utilized to visualize the references that supported the development of in-depth studies. We first performed a coupled network analysis of 292 articles using the VOSviewer software and generated a visual map based on the top 50 citing references with the strongest association strength (**Figure 5A**). Based on coupling strength, these references were classified into four clusters: Cluster 1 (red dominates), consisting of 16 highly citing references with the theme of “Cellular Reprogramming and Regeneration of SPEM”; Cluster 2 (green dominates), consisting of 13 highly citing references with the theme of “The origin of SPEM”; Cluster 3 (blue dominates) consisting of 11 highly citing references, with the theme of “Oxyntic Atrophy, IM and SEPM”, and; Cluster 4 (yellow dominates) comprising a total of 10 highly citing references, with the theme of “Trefoil Factor Family Peptides”. According to the time-overlay visualization map in **Figure 5B**, the references in cluster 1 published by Goldenring (2022) (Goldenring and Mills, 2022), Lee (2022) (Lee et al., 2021), Jeong (2021) (Jeong et al., 2021), Bockerstett (2020) (Bockerstett et al., 2020) and Burclaff (2020) (Burclaff et al., 2020), as well as references in cluster 4 published by Hoffmann (2020 and 2022) (Hoffmann, 2012; Hoffmann, 2020), represent the current frontiers of research. A complete list of the top ten citing references for SPEM-GC is shown in **Table 5**.

**Table 5:** The top 10 citing references based on total link strength in SPEM-GC research.

Rank	Title	First author	Year	Journal	Total link strength	Citations	Cluster	DOI
1	Murine Models of Gastric Corpus Preneoplasia	Christine P. Petersen	2017	Cellular and Molecular Gastroenterology and Hepatology	1468	47	1	10.1016/j.jcmgh.2016.11.001
2	Cellular Plasticity, Reprogramming, and Regeneration: Metaplasia in the Stomach and Beyond	James R. Goldenring	2022	Gastroenterology	1285	7	1	10.1053/j.gastro.2021.10.036
3	Acid and the basis for cellular plasticity and reprogramming in gastric repair and cancer	José B. Sáenz	2018	Nature Reviews Gastroenterology & Hepatology	1226	54	1	10.1038/nrgastro.2018.5
4	Stem Cells, Self-Renewal and Cancer of the Gastric Epithelium	Werner Hoffmann	2012	Curr Med Chem	1086	20	2	10.2174/0929867311209065975
5	Current understanding of SPEM and its standing in the pre-neoplastic process	Victoria G. Weis	2009	Gastric Cancer	1008	87	3	10.1007/s10120-009-0527-6
6	Metaplasia in the Stomach—Precursor of Gastric Cancer?	Hiroto Kinoshita	2017	Int. J. Mol. Sci.	1006	40	1	10.3390/ijms18102063
7	Self-Renewal and Cancers of the Gastric Epithelium: An Update and the Role of the Lectin TFF1 as an Antral Tumor Suppressor	Werner Hoffmann	2022	Int. J. Mol. Sci.	974	0	4	10.3390/ijms23105377
8	Trefoil Factor Family (TFF) Peptides and Their Diverse Molecular Functions in Mucus Barrier Protection and More: Changing the Paradigm	Werner Hoffmann	2020	Int. J. Mol. Sci.	932	25	4	10.3390/ijms21124535
9	Role of metaplasia during gastric regeneration	Emma Teal	2020	American Journal of Physiology-Cell Physiology	901	8	1	10.1152/ajpcell.00415.2019
10	Trefoil factors: Gastrointestinal-specific proteins associated with gastric cancer	Ping Xiao	2015	Clinica Chimica Acta	870	28	4	10.1016/j.cca.2015.08.004

Furthermore, a total of 8431 references were cited at least 23 times based on Price’s law. According to **Table 6**, the ten most co-cited references were cited at least 124 times. One of the most co-cited references was an article by Houghton J et al. published in Science in 2004 (n=903). Nine of the top 10 articles were research papers, and one was a Review. As shown in **Figure 5C**, the reference timeline visualized the evolution of research hotspots over time, and cluster labels were created based on the terms associated with each cluster’s highest frequency. We observed that: cluster #2 (Trefoil factor1), #4 (Gene regulation), #5 (Spasmolytic polypeptide) and #12 (the TTF peptides) started earlier, while cluster #0 (Stem cells), #8 (Differentiation) and #14 (progenitor) could be considered the frontier since they are still ongoing.

**Table 6:** The top 10 globally cited documents based on total citations in SPEM-GC research.

Rank	Title	Document Type	First author	Year	Journal	Citations	DOI
1	Gastric cancer originating from bone marrow-derived cells	*In vivo* study	Houghton J	2004	Science	903	10.1126/science.1099513
2	Reciprocal regulation of gastrointestinal homeostasis by SHP2 and STAT-mediated trefoil gene activation in gp130 mutant mice	*In vivo* study	Tebbutt NC	2002	Nature medicine	382	10.1038/nm763
3	Identification of Molecular Subtypes of Gastric Cancer With Different Responses to PI3-Kinase Inhibitors and 5-Fluorouracil	Clinical research	Lei ZD	2013	Gastroenterology	284	10.1053/j.gastro.2013.05.010
4	GATA-4 and GATA-5 transcription factor genes and potential downstream antitumor target genes are epigenetically silenced in colorectal and gastric cancer	*In vitro* study and clinical research	Akiyama Y	2003	Mol Cell Biol	202	10.1128/MCB.23.23.8429-8439.2003
5	Loss of Klf4 in mice causes altered proliferation and differentiation and precancerous changes in the adult stomach	*In vivo* study	Katz JP	2005	Gastroenterology	183	10.1053/j.gastro.2005.02.022
6	Mature chief cells are cryptic progenitors for metaplasia in the stomach	*In vivo* study	Nam KT	2010	Gastroenterology	176	10.1053/j.gastro.2010.09.005
7	The trefoil factor 1 participates in gastrointestinal cell differentiation by delaying the G1-S phase transition and reducing apoptosis	*In vitro* study	Bossenmeyer-Pourie C	2002	J Cell Biol	146	10.1083/jcb200108056
8	Gastric cancer development in mice lacking the SHP2 binding site on the IL-6 family co-receptor gp130	*In vivo* study	Judd LM	2004	Gastroenterology	141	10.1053/j.gastro.2003.10.066
9	Gastric epithelial stem cells	Review	Mills JC	2011	Gastroenterology	131	10.1053/j.gastro.2010.12.001
10	A molecular signature of gastric metaplasia arising in response to acute parietal cell loss	*In vivo* study	Nozaki K	2008	Gastroenterology	124	10.1053/j.gastro.2007.11.058

Citation bursts are references with a significant increase in citations over time. **Figure 5D** shows the top 25 citation bursts of the 71 detected. The burst with the strongest strength (strength=10.86) was entitled “A molecular signature of gastric metaplasia arising in response to acute parietal cell loss” (Nozaki et al., 2008), published in Gastroenterology by Koji Nozaki et al. in 2008, with citation bursts between 2010 and 2013. Among the 25 references, 10 (40%) were published in 2017-2022, indicating their importance in this field. Notably, 8 (32%) of these 25 papers were still experiencing citation bursts by the time of writing this manuscript. In light of all these factors, we hypothesized that SPEM-GC would continue to attract attention in the future. Additionally, we also found that 5 papers dealt with the proliferation and lineage conversion of SPEM cells (Engevik et al., 2016; Leushacke et al., 2017; Burclaff et al., 2017; Goldenring, 2018; Radyk et al., 2018; Willet et al., 2018) and 3 dealt with mucosal immune regulation (Nozaki et al., 2008; Petersen et al., 2017; Petersen et al., 2018), indicating that SPEM might significantly influence the research field of gastric carcinogenesis by affecting the above mechanisms.

### 3.6 Keywords analysis of trending topic

**Figure 6:**
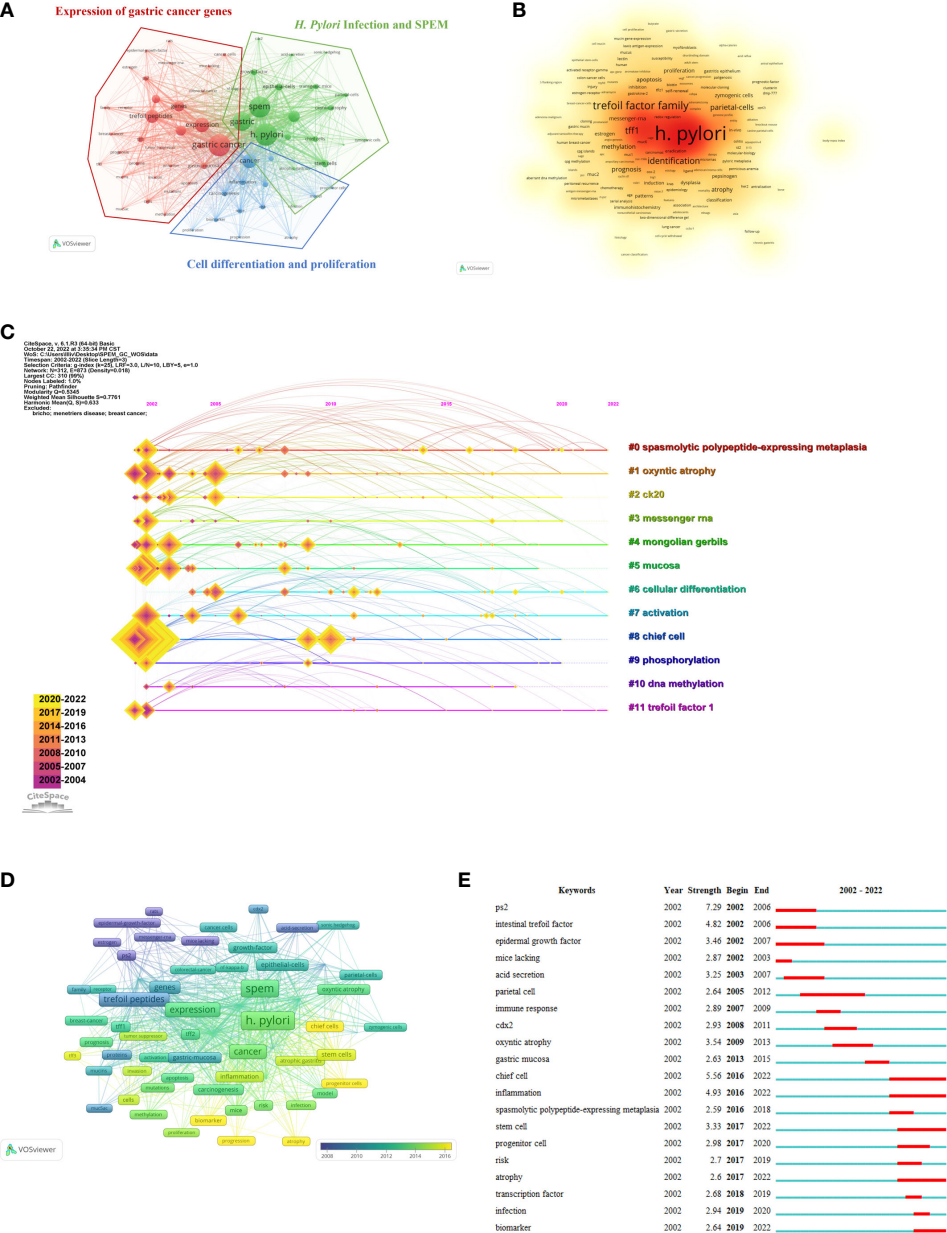
Analysis of trending topics and keywords. **(A)** The co-occurrence keywords with papers ≥ 9 (cluster map). The node’s size represents the frequency of co-occurrence of the keywords, and the link indicates the correlation between keywords. Each node is colored according to the cluster in which the two keywords occur most frequently, with the link thickness being proportional to that number. **(B)** The keywords’ density map drawn using VOSviewer. The word size, roundness and opacity of the orange color are positively correlated with frequency. **(C)** The SPEM-GC timeline view of co-cited keywords. **(D)** Time-overlay visualization map of co-occurrence keywords. **(E)** SPEM-GC’s top 20 keywords with the strongest citation bursts.

A total of 1,394 keywords were extracted from the titles and abstracts of the included papers. Using VOSviewer, 65 keywords appeared more than nine times and were used to generate the visual map in **Figure 6A**. We conducted cluster analysis on high-frequency keywords and divided them into three clusters: Cluster 1 (red dominates), comprising 32 core keywords and was the largest, with the most prevalent keywords being gastric cancer (119 times), expression (79 times), and trefoil peptides (72 times). Cluster 2 (green dominates), comprising 20 core keywords, among which the most common were *H. pylori* (121 times), SPEM (113 times), and gastric (102 times). Cluster 3 (blue dominates), comprising 13 core keywords, with cancer (79 times), inflammation (34 times) and differentiation (30 times) being the most common. **Figure 6B** shows the link strength keywords displayed as a density map, in which the colors indicate the total strength of the links. *H. pylori* (748), gastric cancer (724) and SPEM (706) were the three keywords with the strongest association. **Figure 6C** shows the top 11 high-frequency keywords over time for each cluster. Six out of 11 clusters are still underway. The largest cluster was spasmolytic polypeptide-expressing metaplasia (#0), followed by oxyntic atrophy (#1), Mongolian gerbils (#4), cellular differentiation (#6), activation (#7) and trefoil factor 1 (#11). As a next step, we analyzed the relationship between keywords, the identified themes and three clusters, which is detailed in **Table 7** (Kokol et al., 2022). There were three components to this: expression of gastric cancer genes, *H. pylori* infection and SPEM, as well as cell differentiation and proliferation.

**Table 7:** Clusters of co-cited keywords specialized in SPEM-GC research.

Cluster	Color	Theme	Main frequent codes (total links > 50)	Prevailing sub-categories of clustering analysis
1	Red	Expression of gastric cancer genes	gastric cancer (724), trefoil peptides (470), genes (344), ps2 (178), mucins (103), cancer cells (100), prognosis (97), epidermal-growth-factor (86), invasion (86), methylation (83), activation (79), tumor suppressor (70), messenger-rna (65), mutations (55)	#3 messenger-rna #7 activation #9 phospharylation #10 dna methylation #11 trefoil factor 1
2	Green	*H. Pylori* Infection and SPEM	h. pylori (748), spem (706), gastric (683), intestinal metaplasia (407), epithelial-cells (272), stem cells (251), oxyntic atrophy (208), chief cells (202), parietal-cells (169), atrophic gastritis (114), acid-secretion (93), infection (84), zymogenic cells (79), cdx2 (63), sonic hedgehog (62), nf-kappa-b (59)	#0 spasmolytic polypeptide-expressing metaplasia #1 oxyntic atrophy #5 mucosa #8 chief cell
3	Blue	Cell differentiation and proliferation	cancer (476), carcinogenesis (215), inflammation (206), differentiation (200), identification (178), biomarker (139), progenitor cells (88), progression (70), atrophy (68), proliferation (66)	#1 oxyntic atrophy #5 mucosa #6 cellular differentiation

According to the time-overlay visualization map in **Figure 6D**, “chief cells”, “progenitor cells”, “progression”, “biomarker” and “atrophy” were the recent keywords in addition to keyword trends. Essentially, these keywords seem to outline the current frontiers of research. Additionally, CiteSpace was used to identify the top 20 keywords with the strongest citation bursts (**Figure 6E**). As indicated by the red part, the keywords indicate a blowout at this stage. The keywords related to “ps2” or “trefoil factor 1” bursts were the strongest (strength = 7.29), followed by intestinal “trefoil factor” (strength = 4.82) and “epidermal growth factor” (strength = 3.46). We also found that the keywords were still emerging in 2022. Thus, the research areas of “chief cell”, “inflammation”, “stem cell”, “atrophy” and “biomarker” might become hot spots in the future.

### 3.7 Annotations of SPEM-GC target genes and pathways

**Figure 7:**
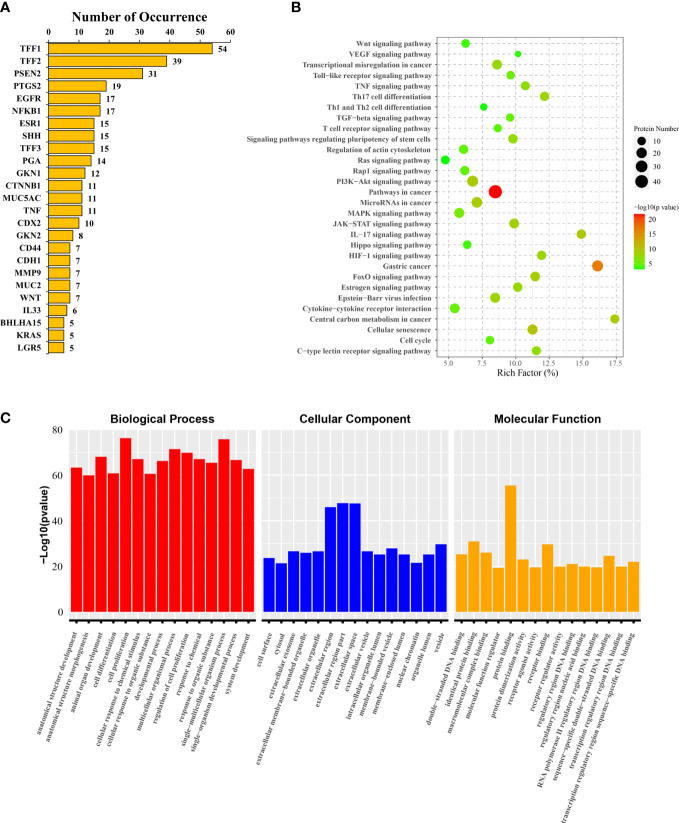
Target genes and pathways annotated for SPEM-GC. **(A)** The 25 high-frequency genes. **(B)** KEGG pathway enrichment analysis results of 147 literature gene keywords. **(C)** GO functional annotation and enrichment analysis results of the literature gene keywords.

To further clarify the focus of mechanistic studies in SPEM-GC-related research fields, we extracted and analyzed the keywords of all associated genes in literature, based on which 147 associated gene keywords were identified. **Supplementary File S2** lists the relevant statistical results. Among them, the most frequently associated gene was TFF1 (54 times), followed by TFF2 (Petersen et al., 2018) and PSEN2 (Nozaki et al., 2008). **Figure 7A** lists the keywords having a frequency >5. KEGG pathway enrichment analysis showed that PI3K-Akt, JAK-STAT, HIF-1, MAPK, Hippo, Wnt, VEGF, cell cycle and Ras signaling pathways were the hot research pathways in the field of SPEM-GC. **Figure 7B** reveals that the immune regulation of Th17 cell differentiation, cytokine-cytokine receptor interaction, and differentiation of Th1 and Th2 cells were also major topics in this field. Further analysis of GO functional annotation and significant enrichment analysis in **Figure 7C** revealed that Biological Process (BP) was predominantly represented in cell proliferation, single-multicellular organism process, and multicellular organismal process. We also found that Cellular Component (CC) was mainly found in the extracellular region, whereas Molecular Function (MF) was mainly found in identical protein binding and receptor binding sites.

## 4 Discussion

### 4.1 General overview

Although SPEM was first reported in 1999 by Schmidts et al. (Schmidt et al., 1999), it was not until 2002 that Halldórsdóttir et al. discovered a link between SPEM and GC (Halldorsdottir et al., 2003). Analysis of the WoSCC database from 2002 to 2022 shows that 292 papers were published in 131 journals by 1757 authors in 412 organizations from 41 nations. Despite relatively stable document volumes, the increasing number of citations indicated that SPEM-GC is becoming increasingly popular. Additionally, SPEM-GC research has steadily grown in the last two decades. According to **Table 2**, the United States contributed to the most number of publications on SPEM-GC among the top ten productive institutions, of which eight were from the United States, one from Japan and one from South Korea. The United States maintained its dominant position in SPEM-GC research with a centrality of 0.47. We also found that Germany, the United States, the United Kingdom and China played key roles in the global collaboration of SPEM-GC research. China was second to the United States as the largest issuer in this field, with a total of 71 issues. Despite the low average citation rate of Chinese scholars (van Eck et al., 2010), compared to the United States (Lei et al., 2013) and Japan (Katz et al., 2005), they have gradually become a central region for research over the past few years (**Figure 2D**).

As one of the top 10 and co-cited authors, James R. Goldenring published the greatest number of SPEM-related papers, indicating his prominent contribution to the field (**Table 3** and **Figure 3A**). The origin of precancerous lesions in the stomach is the primary focus of Dr. Goldenring (Vanderbilt University). Over the past decade, his team has produced paradigm-shifting data proving that precancerous metaplasia does not arise from altered stomach stem cells but develops from the transdifferentiation of protein-secreting chief cells into metaplastic mucous-producing cells (Goldenring et al; Nam et al., 2010; Mills and Goldenring, 2017). They also studied how immune cell populations (M2-macrophages and type II innate lymphoid cells (ILC2s)) modulated the progression of precancerous lesions from metaplasia to growth and proliferative activity (Meyer et al., 2020). Mills Jason C., a Baylor College of Medicine professor, was identified as one of the most productive SPEM-GC authors and ranked in the top 10 and co-cited authors. His research focuses on multiple regulated mechanisms involved in palingenesis during metaplasia and cancer of the gastric lining. In 2022, James R. Goldenring and Jason C. Mills (Goldenring and Mills, 2022). published an important review summarizing SPEM cell regulation mechanisms and pathophysiology in Gastroenterology. According to their study, gastric glands with mixed incomplete intestinal metaplasia and proliferative SPEM might be at greater risk for cancer and dysplasia. In scenarios with chronic pyloric and intestinal metaplasia, targeting early metaplastic lineages could be an effective approach. According to the journal analysis (**Figure 4** and **Table 4**), Gastroenterology published the most SPEM-GC studies, as well as the most cited ones, including 7 of the top 10 highly cited articles (Judd et al., 2004; Katz et al., 2005; Nozaki et al., 2008; Nam et al., 2010; Lei et al., 2013) (**Table 6**). Among the top 3 published journals and top 5 co-cited journals, the American Journal of Pathology and Gut played an essential role in research on SPEM-GC. These journals mainly focus on studies from the Molecular, Biology and Genetics fields. Altogether, these results were consistent with dual-map analysis, which revealed that SPEM-GC research is currently strongly focused on molecular biology and immunology.

In some instances, the knowledge base was represented partly by co-cited references cited by scholars involved in related research. There were four references in the top ten co-cited references related to proliferation, differentiation and precancerous changes within the stomach (Houghton et al., 2004; Katz et al., 2005; Nozaki et al., 2008; Nam et al., 2010), two that elaborated on trefoil factor1 or spasmolytic polypeptides (Bossenmeyer-Pourie et al., 2002; Tebbutt et al., 2002), two on gene regulations (Akiyama et al., 2003; Lei et al., 2013), one on immunoregulation (Judd et al., 2004), and one on the molecular mechanisms of gastric epithelial stem cells (Mills and Shivdasani, 2011). Eight references were still in the burst phase and deserve more attention, among which 5 papers focused on SPEM cell proliferation and lineage transformation (Burclaff et al., 2017; Engevik et al., 2016; Goldenring, 2018; Leushacke et al., 2017; Radyk et al., 2018; Willet et al., 2018) and 3 were on mucosal immune regulation (Nozaki et al., 2008; Petersen et al., 2017; Petersen et al., 2018), indicating that SPEM had a significant impact on the research field of gastric carcinogenesis.

### 4.2 The hotspots and frontiers

For researchers in this age of information explosion and technology, it is essential to keep up with the latest trends in the research field. In bibliometrics, keyword co-occurrences can reflect the focus in specific areas, overlay and timeline views can illustrate the evolution of new hotspots, and the emerging topics in the discipline can be identified through reference clusters and citation bursts (Liu et al., 2022; Li et al., 2022; Zhang et al., 2022a; Yang et al., 2022). As part of this study, we examined reference timeline and burst (**Figures 5C, D**), keyword overlay, co-occurrence, timeline, burst (**Figures 6A–C**), KEGG (**Figure 7B**), and GO annotations (**Figure 7C**) to evaluate the hotspots and frontiers of SPEM-GC research. The 6 hotspots and frontiers of SPEM-GC are discussed below.

#### 4.2.1 Lineage transformation of SPEM cells

A debated topic in SPEM cell development is the lineage transformation of cells. Presently, there are three main hypotheses: “Re-differentiation of chief cells”, “Proliferative of stem cells”, and “Pre-metaplastic cells”. According to Nam et al., SPEM cells can express the chief cell marker Mist1 by cell lineage tracing (Nam et al., 2010). A 2018 study by Radyk et al. reported that tamoxifen induced SPEM cells despite that 5-FU inhibited stem cell proliferation and their location overlapped with the location of chief cells (Radyk et al., 2018). In acute and chronic SPEM animal models, Goldenring et al. (Mills and Goldenring, 2017; Burclaff et al., 2020) found that chief cells in the deep part of gastric mucosal glands could be reactivated for replication. It is thought that mucosal inflammation induced mature differentiated chief cells to convert into SPEM cells, which promoted the repair of the gastric mucosa. However, this process changed the cell structure and transcriptional profiles and increased the expression of proliferative proteins like TFF2, MUC6 and CD44v9. This also increased the possibility of cell carcinogenesis by entering the “cell cycle hit mode”. However, most of the current research on chief cell differentiation relies on animal models, and the hypothesis of SPEM transdifferentiation is still controversial. Hayakawa et al. (Kinoshita et al., 2017) reported in 2017 that the isthmus of the gland might be a major location of acute SPEM proliferation. In contrast, there was little proliferative activity at the gland’s base, which was contradictory to the “Re-differentiation of chief cells” hypothesis. In addition to chief cell redifferentiation, stem cells may also be a source of SPEM cells (Hayakawa et al., 2017). A new hypothesis proposed by this study suggested that SPEM could be a compensatory proliferative process following the loss of parietal cells originating from the isthmus stem cells (Kinoshita et al., 2018). Hata et al. (2020) determined that isthmic stem cells transformed into “precursor SPEM cells” during chronic inflammatory conditions using a G-protein-coupled form of estrogen receptor protein (GPR30) labeling, thus refuting the hypothesis of “Re-differentiation of chief cells”. In 2020 Bockerstett et al. (Bockerstett et al., 2020; Bockerstett et al., 2020) concluded that both stem cells and chief cells could develop into SPEM under 10x single-cell detection. This pre-metaplastic phenotype can be examined in patients with chronic gastritis and SPEM model animals using a quasi-temporal analysis of fundic glandular cells. In addition, SPEMs are hybrid stem/chief cell phenotypes, that is, “Pre-metaplastic cells”.

#### 4.2.2 H. pylori infection and SPEM

By analyzing the relevant literature in the past 20 years, we found that *H. pylori* infection was the most important topic in SPEM-GC research (Meyer and Goldenring, 2018; Yan et al., 2022). A series of changes were reported following *H. pylori* infection, including inflammatory cell infiltration, foveolar hyperplasia, and loss of parietal cells in the gastric mucosa, followed by the appearance of SPEM cells in the mucosa (Yoshizawa et al., 2007). Sanchez et al. (Saenz et al., 2019) research on the host epithelium showed that *H. pylori* could bind to Lewis B (Leb) and Sialyl Lewis X (SLeX) receptors. Tamoxifen combined with *H. pylori* infection induced chronic SPEM in a mouse model by attaching to gastric epithelial cells through blood group antigen-binding Adhesin (BabA) and Sialic acid-binding Adhesion (SabA), respectively. Infection with *H. pylori* promotes the proliferation and progression of SPEM cells, resulting in a vicious cycle (Graham, 2014). Shimizu et al. (2016) reported the development and morphological changes of progressive SPEM glands expressing GSII in a Mongolian gerbil model of *H. pylori* infection in 2016. They found that multiple factors, including host and source of infection, played a role in SPEM caused by *H. pylori* infection. Macrophages, dendritic cells and T cells were induced to bind to *H. pylori* through the p38/ERK1/2 pathway (Krueger et al., 2009; Bernhardt et al., 2010). Studies with genetically engineered mice found that Ctsz and CLDN18 could protect against SPEM (Krueger et al., 2013; Hagen et al., 2018). Besides the host factors discussed above, *H. pylori* virulence factors (e.g., CagA) might also contribute to SPEM development (Wroblewski et al., 2010).

#### 4.2.3 Mucosal microenvironmental disturbances and SPEM

Aside from the above questions on the origin of proliferating SPEM cells, their occurrence and progression are also hot topics in this field. Petersen et al. (2018) illustrated in 2017 how IL-33 might act as an alarm signal to stimulate the type II inflammatory response, thereby driving SPEM development. Parietal cells produce key growth factors, such as dual regulatory proteins, transforming growth factor alpha (TGFα), heparin-binding EGF-like growth factor (HB-EGF) and hedgehog (Shh), which play a key role in SPE proliferation and differentiation (Weis and Goldenring, 2009; Bernhardt et al., 2010; Saenz and Mills, 2018). It was shown in a recent study that the damaged gastric mucosa of SPEM mice and patients with gastric precancerous lesions (GPL) exhibited type II inflammatory reactions and increased the number of ILC2s (Meyer et al., 2020). It was also demonstrated that the suppression of A and B triggered the NF-B/MAPK signaling pathway in GATA3+ ILC2s, inducing aggravating mucosal immune damage and allowing SPEM to develop by upregulating type II cytokines, including IL-13, IL-4, IL-5 and IL-9 (Buzzelli et al., 2015; Liu et al., 2015; Bando and Colonna, 2020). Also, IL-13 released after acute parietal cell injury activated macrophages into M2 macrophages and contributed to SPEM production (De Salvo et al., 2021). It is crucial for gastric mucosal cells to have M2 macrophages to upregulate SPEM and IM-related genes, such as trefoil factor 3 (TFF3), cystic fibrosis transmembrane regulator (CFTR) and deleted in malignant brain tumors 1 (DMBT1), during metaplasia progression (Choi et al., 2016; Meyer and Goldenring, 2018). Thus, inhibiting the IL-33 or IL-13 cytokine pathway could regulate macrophage polarization and be considered for potentially treating GPL (Hayashi et al., 2012). In addition, it has been found that the expression of structurally activated Kirsten rat sarcoma viral oncogene (Kras) in chief cells could promote M2 macrophage infiltration in the gastric mucosa, aggravating the development of SPEM (Choi et al., 2016; Goldenring and Mills, 2022). Previous studies have demonstrated that chronic inflammation cytokines TLR9 and IFN-γ directly induce apoptosis in gastric epithelial cells. They also developed chronic atrophic gastritis and SPEM, subsequently increasing their risk of SPEM-related carcinogenesis (Osaki et al., 2019; Ding et al., 2022).

#### 4.2.4 Progression and outcome of SPEM

The progression and outcome of SPEM lesions is also a controversial topic. By clarifying the relationship between SPEM and gastric cancer and precancerous lesions, the nature of SPEM lesions can be accurately defined and then matched clinical prevention and treatment. The accumulation of mutations in long-lived mature cells can be viewed in basic research. Chief cells found in areas of initial metaplasia foci were found to be the most damaged. Upon accumulating a certain amount of mutations, SPEM cells evolve into clonal forms of expansion, becoming the cell of origin of dysplasia and even gastric cancer (Saenz and Mills, 2018; Miao et al., 2021). Clinical study results from Singapore showed that MUC5AC, KRAS, BRAF and EZH2 mistranslated mutations were more prevalent in SPEM, which are more genetically similar to GC tissues (Srivastava et al., 2020). Another retrospective cohort study from Iceland (Halldorsdottir et al., 2003) reported 82% of preoperative gastric mucosal biopsy specimens tested positive for SPEM, which is significantly higher than IM (57%). Additionally, SPEM models constructed from feline *H. pylori*-infected mice were found to have more DNA mismatch repair gene defects than normal gastric glands, consistent with genetic analyses of precancerous tissues (Wang et al., 2014). During gastric carcinogenesis, SPEM glands exhibit genetic instability because the genetic properties of the source stem cells can accumulate enough mutations to cause GC (Chen et al., 2020). The above results provide indirect evidence that SPEM increases the risk of carcinogenesis in precancerous tissues. Nevertheless, some scholars, such as Graham et al. (Graham and Zou, 2018), concluded that prior studies on SPEM’s progression to GC were inconsistent, with limited clinical evidence based on human tissues. Additionally, autoimmune gastritis can also lead to SPEM with or without IM, whereas it rarely progresses to GC.

#### 4.2.5 Clinical diagnosis and biomarkers for SPEM

SPEM is also known as pseudopyloric gland metaplasia, mucinous metaplasia or corpus antrum metaplasia (Schmidt et al., 1999; Saenz and Mills, 2018; Goldenring, 2018). SPEM lesions usually occur in the deep part of the glandular duct in the early stages of human gastric mucosa injury, making endoscopic diagnosis difficult. Thus, discovering more sensitive, specific and simple detection methods paves the way for subsequent clinical interventions. Previous studies found that SPEM predominantly co-expressed TFF2 and MUC6 (Bockerstett et al., 2020), and since then, more relevant biomarkers have emerged over time. A corpus-predominant gastritis index (CGI) was proposed by Tsai et al. (2013) in 2013. According to relevant clinical studies, the incidence of SPEM in CGI-positive patients was significantly higher than in CGI-negative, suggesting that it can be used as an early detection tool for precancerous lesions (Cheng et al., 2017). As a follow-up to the above studies, Kuo et al. examined the association between serum TFF2 levels and the expression of miR-21, 155 and 223 in gastric mucosa for SPEM and reported that the above molecules might have diagnostic values (Kuo et al., 2017; Kuo et al., 2019). Subsequent studies successively showed that GSII (Shimizu et al., 2016), CD44v9 (Bertaux-Skeirik et al., 2017; Zavros, 2017), Clusterin (Vange et al., 2017), SRY-related high mobility group box gene 9 (SOX9) (Serizawa et al., 2016), human epididymis protein 4 (HE4) (Nozaki et al., 2008; Jeong et al., 2021) and myelin and lymphocyte protein 2 (MAL2) (Weis et al., 2014) were associated with the expression and proliferation of SPEM cells. In recent studies, aquaporin 5 (AQP5), Trop2 and DDIT4 were shown to reflect parietal cell loss and the severity of SPEM development and could also predict a higher risk of GC (Riera et al., 2020; Lee et al., 2021; Miao et al., 2021).

#### 4.2.6 Construction of proliferative SPEM model animals

Establishing a sustained and stable animal model of proliferative SEPM is the basis for studying the pathogenesis of SPEM. Since SPEM pathological evolution is a chronic pathogenetic process, corresponding methods like *H. pylori*-infected model take months to complete, which is not conducive to studying gastritis and GC in humans (Li et al., 2021). Animal models of acute SPEM induced by chemical drugs are currently being used as an attempt to shorten modeling time, including DMP-777 and L635, as well as the intraperitoneal injection of tamoxifen, a selective estrogen receptor modulator at high dose (Petersen et al., 2017). A loss of parietal cells can be caused by any of the three methods, resulting in the formation of SPEM at the stomach base of mice. DMP-777 is a cell-specific inhibitor of neutrophil elastase that destroys parietal cells without causing inflammatory reactions. Unlike DMP-777, L635 exerts similar inflammatory effects to H. Felis infection without elastase inhibitors, but it is expensive and difficult to obtain. Similarly, reversible acute parietal cell injury was observed in mice from high tamoxifen doses, but this model did not cause gastric inflammation and recovered within three weeks (Saenz et al., 2016; Lee et al., 2017; Meyer et al., 2020). It must be noted that although the above drugs can induce SPEM, the observed metaplasia is reversible and does not match the chronic SPEM development in clinical situations. Hence, further research is needed to explore a research model similar to chronic SPEM in clinical settings.

### 4.3 Strengths and limitations

Overall, this present study represents the first bibliometric analysis of SPEM-GC-related publications in the past two decades. The presented type of analysis offers a fresh and objective perspective on evolving research topics and trends, which cannot be obtained through traditional reviews. As part of the research, a multidimensional analysis was conducted using various bibliometric software tools to provide more comprehensive results for readers. As a comprehensive guide for future developments in GC research, this study also provides scholars with an overview on SPEM-GC research and an objective guide for the public to understand the significance of SPEM. However, there were some limitations in this study. First, as only WoSCC data were used, it is possible that some relevant studies in PubMed, Scopus and other databases were excluded, resulting in incomplete data collection. However, it should be noted that WoSCC indexes the largest number of articles, ensuring source integrity. Additionally, high-quality articles published in other languages were not considered due to the focus on English-only articles. Lastly, due to methodological limitations of the overall literature quality evaluation system, some newly published high-quality documents might not have been included in the bibliometrics analysis due to their low citations and centrality.

### 4.4 Conclusion

We report the first bibliometric analysis summarizing the knowledge map of research between SPEM and gastric cancer in the last two decades and the potential future research hotspots. We found that the United States had the most high-quality publications and the greatest international cooperation and communications. However, worldwide collaboration among organizations needs to be improved. Additionally, although SPEM could be identified through multiple biomolecular markers as a possible source of GC, there is still a lack of understanding on how SPEM contributes to the early detection and treatment of cancer, indicating a potential research area and discoveries in the future. The findings of this study can be used as a guide for choosing research topics, defining the most promising research frontiers, and selecting appropriate journals for publication. Additionally, we provided information to clinicians and practitioners on new approaches and technologies that might enhance the treatment of H. pylori infection-related gastric mucosal diseases and benefit populations at high risk of developing GC.

## Data availability statement

The original contributions presented in the study are included in the article/**Supplementary Material**. Further inquiries can be directed to the corresponding author.

## Author contributions

XDT designed this study and revised the paper. LL, YW and YKZ conducted data analysis and paper writing. LL and WZ performed the bibliometric analyses. JL, PW and FYW made the figures and tables. All authors contributed to the article and approved the submitted version.
